# Supplementary material for: Prospective Comparison of Nine Different Handheld Ultrasound (HHUS) Devices by Ultrasound Experts with Regard to B-Scan Quality, Device Handling and Software in Abdominal Sonography
Source: Diagnostics (Basel). 2024 Aug 30;14(17):1913. doi: 10.3390/diagnostics14171913 (PMC11393954; doi:10.3390/diagnostics14171913)
Supplement: Supplementary file 1 [file diagnostics-14-01913-s001.zip › diagnostics-3158066-supplementary.pdf]

Supplement

Table S1. Baseline characteristics of the 12 examiners.

Item	Category	n	%
Discipline	Internal medicine	8	67
	Intensive care	2	17
	Oncology	1	8
	Radiology	1	8
Experience (years)	> 3	1	8
	> 5	2	17
	> 10	9	75
Ultrasound examinations: total performed	> 3000	6	50
	> 2000	4	33
	> 1000	2	17
Ultrasound examinations: per week	> 50	4	33
	> 30	7	58
	> 20	1	8
HHUS experience	Yes	3	25
	No	9	75
DEGUM level	none	6	50
	I	1	8
	II	5	42
	III	1	8
Present medical setting	In-patient	7	58
	Out-patient	5	42

Table S2. Grading of HHUS devices overall, per main item overall and per subitem shown as median and IQR.

Item		Butterfly iQ+	Clarius C3HD3	Sono-Site iViz	Philips Lumify	D5CL Microvue	SonoEye Chison	TE Air Mind-ray	Vscan Air GE	Youkey Q7
All items		4 (4, 3)	4 (4, 3)	3 (4, 2)	4 (4, 3)	4 (4, 3)	4 (5, 4)	3 (4, 3)	4 (5, 4)	4 (4, 3)
B-mode image quality	**Overall**	3 (4, 2.25)	4 (5, 4)	3 (4, 3)	3.5 (4, 3)	3 (4, 3)	4 (4, 3)	3 (3, 2)	4 (5, 4)	4 (4, 3)
	Resolution	3 (4; 2)	4 (5; 4)	3 (4; 2.25)	4 (4; 3)	3.25 (4; 2.5)	3 (4; 2)	4 (4; 3.25)	3 (4; 2)	4 (4; 3)
	Contrast	3 (3.75; 2.25)	4 (5; 4)	3 (4; 3)	3 (4; 3)	3.25 (4; 3)	4 (4.75; 3.25)	3 (3.75; 3)	4 (4; 3.25)	4 (4; 3)
	Overall impression	3 (3.75; 3)	4 (5; 4)	3.5 (4; 3)	4 (4; 3.25)	4 (4; 3)	3 (4; 3)	4 (4.75; 3.25)	4 (4; 3.25)	4 (4; 3)

Handling	**Overall**	4 (4, 3)	4 (4, 3)	3 (4, 3)	4 (4, 3)	4 (5, 4)	4 (4, 3)	4 (4, 3)	4 (5, 3)	4 (5, 3)
	Haptics	3.5 (4; 3)	3 (4; 3)	3.5 (4; 3)	4 (5; 4)	4 (5; 4)	4 (5; 3)	4 (4.75; 3)	4 (4; 3.75)	3 (4; 3)
	Weight	3 (3.75; 3)	3 (4; 2)	4 (4; 3)	4 (5; 4)	4 (5; 4)	4 (5; 3.25)	4 (4; 3)	4 (4; 3.25)	4 (4; 3)
	Shape	4 (4; 3)	3 (4; 3)	3 (4; 3)	4 (4; 3.25)	4 (4; 3)	5 (5; 4)	3 (4; 2.25)	3 (4; 3)	4 (4; 3)
	Connectivity	4 (4; 4)	4 (4; 3.25)	3.5 (4; 3)	3 (4; 3)	3.25 (4; 3)	4 (4.75; 3.25)	4 (5; 3.25)	4 (4; 3)	4 (4; 4)
	Overall impression	3.5 (4; 3)	4 (4; 3)	3 (4; 2.25)	4 (4; 3.25)	4 (4; 3.25)	4 (4; 3)	4 (4; 3.75)	4 (4; 4)	4 (5; 3)
Software	**Overall**	4 (4, 3)	4 (4, 3)	3 (3, 2)	4 (4, 3)	3 (4, 3)	4 (5, 4)	3 (4, 3)	4 (5, 4)	4 (4, 3)
	Presets	4 (4; 3)	4 (4; 4)	2.5 (3; 2)	4 (4; 3)	4 (4.75; 4)	4 (4; 3)	4 (4.75; 3)	4 (4; 3)	4 (4; 3)
	Depth	4 (4; 4)	4 (4; 3.25)	2.5 (3; 2)	4 (4; 3.5)	4.5 (5; 3.25)	3 (4; 2)	4 (5; 3.5)	4 (4; 3.25)	3.5 (4; 3)
	Gain	4 (4; 4)	4 (4; 3.25)	3 (3; 2)	4 (4; 3)	4 (5; 4)	3 (4; 3)	4 (5; 4)	4 (4; 3.75)	4 (4; 3)
	Duplex/PRF	4 (4; 3)	3 (4; 3)		3 (4; 3)	4 (5; 4)	3 (4; 3)	4 (5; 4)	4 (4; 3)	4 (4; 3)
	Saving	4 (4; 3.25)	4 (4.75; 3)	3 (4; 2)	4 (4; 3)	4 (4; 3.5)	4 (4; 3)	5 (5; 4)	4 (4; 3.25)	4 (4; 3)
	Intuitiveness	4 (4; 3)	4 (4; 3)	3 (3; 2)	3.5 (4.75; 3)	4 (5; 3.5)	4 (4; 3)	3.5 (4; 3)	4 (4; 3)	4 (4; 3)
	Overall impression	4 (4; 3)	4 (4; 3)	2 (3; 2)	4 (4; 3)	4 (4; 3)	3 (4; 3)	3.75 (4; 3)	4 (4; 3)	4 (4; 3)

Table S3. P-values of the comparisons between the individual HHUS devices regarding B-mode image quality (a), device handling (b), software (c) and overall grade (d). P < 0.05 marked in green.

(a) B-mode image quality

	Clarius C3HD3	Vscan Air	Chison Sonoeye	Youkey Q7	Philips Lumify	SonoSite iViz	Microvue D5CL	Butterfly iQ+
Vscan Air	0.08							
Chison Sonoeye	0.067	0.09						
Youkey Q7	0.002	0.004	0.26					
Philips Lumify	< 0.001	< 0.001	0.004	0.99				
SonoSite iViz	< 0.001	< 0.001	< 0.001	0.3	0.3			
Microvue D5CL	< 0.001	< 0.001	< 0.001	0.29	0.29	0.08		
Butterfly iQ+	< 0.001	< 0.001	< 0.001	0.023	0.023	0.23	0.31	
Mindray TE Air	< 0.001	< 0.001	< 0.001	< 0.001	< 0.001	< 0.001	< 0.001	0.02

(b)

Device handling

	Microvue D5CL	Youkey Q7	SonoSite iViz	Vscan Air	Chison Sonoeye	Butterfly iQ+	Mindray TE Air	Clarius C3HD3
Youkey Q7	0.011							
Vscan Air	0.001	0.59						
Philips Lumify	< 0.001	0.3	0.62					
Chison Sonoeye	< 0.001	0.01	0.12	0.25				
Butterfly iQ+	< 0.001	0.06	0.02	0.05	0.61			
Mindray TE Air	< 0.001	0.04	0.01	0.03	0.60	0.97		
Clarius C3HD3	< 0.001	< 0.001	0.002	0.006	0.22	0.47	0.8	
SonoSite iViz	< 0.001	< 0.001	< 0.001	< 0.001	0.14	0.33	0.33	0.81

(c)
Software

	Chison Sonoeye	Vscan Air	Butterfly iQ+	Clarius C3HD3	Philips Lumify	Youkey Q7	Mindray TE Air	Microvue D5CL
Vscan Air	0.37							
Butterfly iQ+	< 0.001	< 0.001						
Clarius C3HD3	< 0.001	< 0.001	0.78					
Philips Lumify	< 0.001	< 0.001	0.12	0.22				
Youkey Q7	< 0.001	< 0.001	0.17	0.28	0.92			
Mindray TE Air	< 0.001	< 0.001	< 0.001	0.003	0.07	0.07		
Microvue D5CL	< 0.001	< 0.001	< 0.001	< 0.001	0.06	0.05	0.96	
SonoSite iViz	< 0.001	< 0.001	< 0.001	< 0.001	< 0.001	< 0.001	< 0.001	< 0.001

(d)
Overall grade

	Vscan Air	Chison Sonoeye	Clarius C3HD3	Youkey Q7	Philips Lumify	Microvue D5CL	Butterfly iQ+	Mindray TE Air
Chison Sonoeye	0.66							
Clarius C3HD3	< 0.001	< 0.001						
Youkey Q7	< 0.001	< 0.001	0.97					
Philips Lumify	< 0.001	< 0.001	0.29	0.29				
Microvue D5CL	< 0.001	< 0.001	0.41	0.44	0.93			
Butterfly iQ+	< 0.001	< 0.001	0.018	0.018	0.19	0.19		
Mindray TE Air	< 0.001	< 0.001	< 0.001	< 0.001	< 0.001	< 0.001	< 0.001	
SonoSite iViz	< 0.001	< 0.001	< 0.001	< 0.001	< 0.001	< 0.001	< 0.001	0.016

Table S4. Intraclass Correlation (ICC) based on the 3 main subjects and 12 raters. ICC type 3,k as referenced by Shrout & Fleiss (1979).

**Device**	**Point Estimate (Lower and upper 95% CI)**
Butterfly iQ+	0.86 (0.37 – 1.00)
Clarius C3HD3	0.92 (0.64 – 1.00)
D5CL Microvue	0.95 (0.77 – 1.00)
Philips Lumify	0.73 (-0.18 – 0.99)
SonoEye Chison	0.90 (0.55 – 1.00)
SonoSite iViz	0.90 (0.57 – 1.00)
TE Air Mindray	0.93 (0.70 – 1.00)
VScan Air GE	0.77 (-0.01 -0.99)
Youkey Q7	0.83 (0.25 – 1.00)
